# Nationwide assessment of community pharmacists’ practices and atorvastatin–drug interactions in Egypt

**DOI:** 10.1038/s41598-026-40872-1

**Published:** 2026-03-27

**Authors:** Mohammed G. Maslub, Mahasen Ali Radwan, Nur Aizati Athirah Daud, Zeyad Ali Abd-Alla, Marwa Adham El-Mohamdy

**Affiliations:** 1 Department of Clinical Pharmacy and Pharmacy Practice, Faculty of Pharmacy, Egyptian Russian University, Cairo, 11829 Egypt; 2 School of Pharmaceutical Sciences, Universiti Sains Malaysia, USM, 11800 Pulau, Pinang Malaysia; 3 Human Genome Centre, School of Medical Sciences, Universiti Sains Malaysia Health Campus, 16150 Kota Bharu, Keclantan Malaysia; 4 Department of Clinical Pathology, Faculty of Medicine, Ain Shams University, Cairo, 11591 Egypt

**Keywords:** Atorvastatin, Drug–drug interactions, Community pharmacists, Pharmacovigilance, Clinical practice, Medication safety, Diseases, Drug discovery, Health care, Medical research

## Abstract

Atorvastatin (ATV) is widely prescribed in Egypt, where cardiovascular disease remains the leading cause of mortality; however, it is prone to clinically significant drug–drug interactions (DDIs). This national cross-sectional study evaluated community pharmacists’ (CPs’) knowledge, practice behaviors, and reported rates of ATV-related DDIs across Egypt. A total of 973 licensed pharmacists completed a validated survey. The mean cumulative practice score was 7.2 ± 1.91, indicating high adherence to recommended safety practices. Frequently reported roles included targeting polypharmacy patients (80.1%), initiating single-drug therapy when appropriate (82.2%), counseling patients about adverse effects (85.1%), and using web-based drug-information resources (88.2%), whereas structured DDI screening tool use was less common (46.1%). Compared with male pharmacists, female pharmacists presented significantly higher cumulative practice scores (7.52 ± 1.59 vs. 7.08 ± 1.99; *p* = 0.016), and diploma holders scored higher than Ph.D. holders did (7.47 ± 1.81 vs. 6.82 ± 1.87; adjusted *p* = 0.02). High-frequency encounters were reported for major DDIs with cyclosporine (49.3%) and clarithromycin (45.8%) and for moderate DDIs with digoxin (42.2%), phenytoin (41.0%), and azithromycin (40.5%). Regional variation showed higher interaction frequencies in Upper Egypt and the Delta. Strengthening pharmacist stewardship programs and expanding standardized DDI screening approaches may improve ATV safety.

## Introduction

Cardiovascular disease (CVD) remains the leading cause of mortality in Egypt, accounting for nearly half of all deaths^1,2^. Dyslipidemia is a major modifiable contributor to CVD, and its high national burden is well documented, with approximately 37% of Egyptians reporting elevated blood cholesterol levels^2–4^. Hydroxymethyl glutaryl-CoA (HMG-CoA) reductase inhibitors are the cornerstone of dyslipidemia management and have been shown to substantially reduce CVD risk^5^. Interindividual variability in statin response and tolerability is increasingly recognized and has been linked to pharmacogenetic differences affecting drug transport and metabolism^6,7^. In Egypt, statin monotherapy is the predominant lipid-lowering therapy, with ATV being the most widely prescribed agent^8^.

Although clinically effective, ATV has a well-recognized risk of adverse effects, including myopathy^5,9,10^ and elevated hepatic transaminases^5,11,12^. The estimated incidence of myopathy among statin users ranges from 0.1 to 0.2%^5^; however, myotoxicity represents the leading reason for statin discontinuation^5,13,14^. Mechanistically, statin-associated myopathy is linked to direct inhibition of the HMG-CoA reductase enzyme^5^, and this risk is markedly amplified in the presence of DDIs. Approximately 60% of statin-induced rhabdomyolysis episodes are attributed to DDIs^5,15^. Clinically, ATV-DDIs can significantly increase systemic ATV exposure, primarily through the inhibition of metabolizing enzymes and drug transporters such as cytochrome P450 isoenzymes and uptake/efflux pathways^7,16^.

Pharmacists are central to preventing drug-related problems (DRPs) in routine care^17,18^. Their comprehensive understanding of medication therapy positions them to identify and resolve safety issues, including DDIs, before they lead to adverse outcomes^18^. Evidence consistently shows that pharmacist-led medication reviews reduce DRPs^19^, decrease adverse drug event risk^19,20^, and minimize polypharmacy and inappropriate medication use^19^. CPs, in particular, are strategically positioned to identify polypharmacy-related concerns^21^ and to provide essential patient education—an integral component of chronic disease management and treatment optimization^22–24^.

Given the complexity and clinical importance of DDIs, pharmacists frequently rely on electronic DDI checkers^25,26^. However, the variability in DDI classification across platforms necessitates the use of more than one resource to ensure accuracy and comprehensive detection^27–29^. These digital tools play crucial roles in supporting clinical decision-making and improving patient safety^27^.

Despite the high prevalence of dyslipidemia in Egypt^2–4^ and the widespread use of ATV, there is a notable lack of research evaluating the knowledge and practices of CPs regarding ATV-related DDIs. The international literature underscores the importance of guideline-directed dyslipidemia management and the risks associated with suboptimal adherence^1,2^; however, no national studies have examined whether pharmacists can identify clinically relevant ATV-DDI patterns that may signal suboptimal adherence to prescribing guidelines, patient-level risk factors, or regional care disparities.

This study addresses this gap by assessing pharmacists’ knowledge of clinically significant ATV-DDIs, their practice behaviors in preventing DRPs within community settings, and their reported rates of key ATV-DDIs in daily practice. Understanding CPs’ perceptions and real-world observations may provide indirect insights into prescribing quality, patient counseling needs, and the overall safety of dyslipidemia management in Egypt. These findings may further contribute to ongoing international efforts to strengthen statin safety monitoring and enhance pharmacist-led interventions in cardiovascular risk reduction.

## Methods

### Study design

A descriptive, cross-sectional survey was developed and distributed online. The study adhered to the Declaration of Helsinki and received ethical approval in October 2021 from the Scientific Research & Ethics Committee of the Faculty of Pharmacy, Egyptian Russian University, Cairo, Egypt (reference no. ECH-026).

### Study participants

Eligible participants were licensed pharmacists practicing in community pharmacies across Egypt’s 27 administrative governorates. For analytical purposes, governorates were grouped into five geographic regions in accordance with commonly used national administrative classifications. The Greater Cairo region included Cairo, Giza, and Qalyubia; the Alexandria region included Alexandria, Beheira, and Matrouh; the Delta region included Dakahlia, Monufia, Gharbia, Kafr El-Sheikh, and Damietta; the Canal region included Sharqia, Ismailia, Suez, Port Said, and North and South Sinai; and Upper Egypt included Fayoum, Beni Suef, Minya, Assiut, New Valley, Sohag, Qena, Luxor, Aswan, and the Red Sea. Participation was voluntary, and participants were free to withdraw from the study at any time without penalty.

### Sample size

A total sample size of 384 pharmacists was calculated via Cochran’s formula on the basis of a population of 216,072 licensed pharmacists in Egypt (23 pharmacists per 10,000 of the population)^30^, a 5% margin of error, a 95% confidence interval, and a standard deviation of 0.5^31^.

### Cover letter and consent procedures

Each participant received a cover letter outlining the study’s purpose and objectives, the voluntary nature of participation, assurances of confidentiality, the estimated time required to complete the survey, and the investigators’ contact information. Consent for both participation and publication was obtained verbally or through implied consent via survey completion, according to participant preference.

### Instrument development

The questionnaire was developed and administered in English, which is the primary language of pharmacy education and professional practice in Egypt. The survey instrument was designed following a structured, multistage approach:

#### Item generation

Domains were identified on the basis of earlier studies on DRPs, DDIs, and statin safety^5,15–21,25–29,32–40^. Dyslipidemia guidelines were consulted to ensure clinical relevance^1,2^.

#### Expert review

A panel of five experts (two clinical pharmacists, two academic researchers, and one cardiologist) reviewed the content for clarity, completeness, and relevance. On the basis of their recommendations, the following changes were made: removal of redundant items; rewording of ambiguous questions; addition of one knowledge-based item related to pharmacokinetics; and refinement of Likert-scale response anchors for DDI frequency, including agreement on operational definitions of frequency categories.

#### Pilot testing

A pilot study with 15 pharmacists confirmed the clarity and feasibility of the study, which required no major revisions.

### Reliability and validity

Internal consistency was evaluated via Cronbach’s alpha (α = 0.843). A value of α ≥ 0.70 was considered acceptable.

### Survey structure

The questionnaire comprises three domains: (I) demographics; (II) practice behaviors, including 10 items assessing pharmacists’ implementation of key DDI prevention practices; and (III) the observed frequency of ATV-related DDIs, categorized as major or moderate according to IBM Micromedex® classifications. A cumulative practice score (range: 0–10) was calculated, with higher scores indicating greater adherence to recommended pharmacist behaviors. The survey used closed-ended, mutually exclusive nominal or ordinal response formats. An open-ended item at the end of the survey invited respondents to report any minor ATV-related interactions and provide additional comments.

The first domain collected demographic characteristics, including name, gender, age, contact information, governorate, highest educational degree, years of practice as a CP, average daily patient volume, and the primary information resource used to remain informed about potential DDIs.

The second domain consisted of 10 dichotomous items assessing CPs’ routine engagement in practices that support the detection of potential ATV-related DIs. Respondents indicated “yes” or “no” for the following practices: (1) reviewing all patient medications^32,33^; (2) experience in managing patients with polypharmacy^34,35^; (3) initially endorsing nonpharmacological treatment approaches when appropriate^35,36^; (4) initiating therapy with a single medication when feasible^32,36^; (5) educating patients about potential ATV-related DIs^32,36^; (6) encouraging patients to read OTC medication labels for potential interactions with ATV^32^; (7) inquiring about personal or family history of ATV or other statin use^32^; (8) utilizing web-based, practitioner-oriented resources (e.g., Medscape, Drugs.com, FDA.gov) to obtain up-to-date, reliable drug information^37^; (9) using screening tools such as the Drug Associated Risk Tool (DART) to identify at-risk patients^32,38^; and (10) possessing knowledge of the pharmacokinetics of ATV and concomitantly used medications^32,39^. A cumulative score was generated for each participant, assigning 1 point for “Yes” and 0 points for “No.”

The third domain assessed the frequency with which CPs encountered ATV-related DIs. Respondents were presented with a list of medications and asked to rate the frequency of interactions via a 5-point Likert scale: never (0), rarely (1), sometimes (2), often (3), and always (4). For standardization, the categories were operationally defined as follows: “never” indicated that the pharmacist had not encountered the interaction in routine practice; “rarely” indicated encounters occurring infrequently or sporadically (e.g., less than once per month); “sometimes” indicated occasional encounters (e.g., approximately 1–3 times per month); “often” indicated regular encounters (e.g., approximately once per week); and “always” indicated very frequent encounters occurring in most routine practice weeks. These responses were subsequently recoded into low-frequency (0), intermediate-frequency (1–2), and high-frequency (3–4) categories, which is consistent with recommended approaches for transforming ordinal Likert-type survey data into analytically meaningful grouped categories to enhance interpretability and statistical applicability^40^. Interactions were grouped into “major” and “moderate” categories on the basis of the IBM Micromedex® definitions: a “Major DDI” is “life-threatening and/or requires medical intervention to minimize or prevent serious adverse events,” whereas a “moderate DDI” “may result in an exacerbation of the patient’s condition and/or require an alteration in therapy”^41^. IBM Micromedex® and Drugs.com were selected as reference sources because of their comparable performance in detecting potential drug–drug interactions (pDDIs)^42^. Medications were included if they met three predefined criteria: (1) consistent classification under the same severity category across multiple DDI databases to improve the reliability of severity categorization^42^; (2) established clinical significance on the basis of documented pharmacokinetic or pharmacodynamic mechanisms known to alter ATV exposure or toxicity risk^5,7,16^; and (3) expected relevance to routine community-pharmacy practice on the basis of their documented use in common comorbid conditions^43–52^. Only medications consistently classified under the same severity category in both databases were included. Ten medications constitute the “major” domain: clarithromycin, cyclosporine, erythromycin, fenofibrate, fluconazole, gemfibrozil, ketoconazole, leflunomide, nefazodone, and niacin^41,53^. Seven medications are categorized as “moderate”: amiodarone, digoxin, azithromycin, pectin, clopidogrel, phenytoin, and fluvoxamine^41,53^.

### Data collection

Google Forms were used to prepare the survey and collect responses, and the form was set to accept only one response per participant. The link to the survey form was shared via social media platforms, email, or text messages. The survey was open for responses from 1 May 2023 to 1 January 2025. To ensure response authenticity and reduce the risk of duplicate entries into the form via different devices or emails, the participants were requested to print and fill out the questionnaire in writing, stamp the document via the pharmacy’s stamp, and then scan and upload the image in the form response. A complete submission was defined as a response in which participants completed the survey and provided sufficient data for primary outcome analyses. Isolated missing responses within demographic variables were retained and analyzed via available-case analysis.

### Statistical analysis

The data were analyzed via the IBM Statistical Package for Social Sciences (SPSS) for Windows, version 27 (IBM Corp., Armonk, NY, USA). Descriptive statistics are presented as frequencies and percentages for categorical variables and as the means ± standard deviations (SDs) for continuous variables. Associations between categorical variables and demographic characteristics were assessed via Pearson’s chi-square test of independence.

The distribution of continuous variables, including cumulative role scores, was assessed via the Shapiro–Wilk test, which is recommended for evaluating normality in biomedical datasets. As cumulative role scores demonstrated statistical deviation from normality, nonparametric statistical methods were applied for inferential group comparisons. Specifically, differences between two groups were assessed via the Mann–Whitney U test, whereas comparisons across three or more groups were performed via the Kruskal–Wallis H test.

Despite the observed deviation from normality, continuous variables are presented as the means ± SDs due to the large overall sample size (n = 973)^54^. In large samples, the sampling distribution of the mean approximates normality according to the Central Limit Theorem, allowing mean-based descriptive statistics to remain interpretable even when raw data are not normally distributed^54–56^. This reporting approach is widely accepted in biomedical and epidemiological research, particularly in large observational studies^54,56^. Statistical significance was defined as a two-sided *P* value ≤ 0.05.

## Results

### Response rate and sample characteristics

A total of 1,444 responses were collected. After excluding duplicate entries (n = 186), incomplete submissions (n = 213), and ineligible participants who were either not licensed CPs or not practicing in Egypt (n = 72), 973 valid responses were included in the final analysis. Although the minimum required sample size was 384, all eligible responses were retained for analysis to improve precision and statistical power. The participants’ demographic characteristics are summarized in Table 1. Most respondents were male (73.0%) and within the 20–39-year age range (69.0%). Approximately 66.7% held a bachelor’s degree, and 31.4% had 5–10 years of professional experience. Pharmacies serving 50–100 patients per day constituted the largest group (38.8%). Internet or mobile applications were the most commonly used resources for checking drug–drug interactions (54.5%), followed by medical textbooks (29.6%). Across regions, internet/mobile applications were the most commonly used DDI resource, ranging from 44.7% in Upper Egypt to 61.6% in Greater Cairo. The full demographic distributions are presented in Table 1.

**Table 1:** Demographics, pharmacists’ roles, and major or moderate DDIs (n = 973).

Demographics (n = 973)		CP’s roles to check pDDIs (n = 973)			Reported rates of ATV-related DDIs (n = 973)			
Age	n (%)		Yes n (%)	No n (%)	i. Major	n (%)	ii. Moderate	n (%)
20–29 years	333 (34.2)	Cumulative roles score*	7.2 ± 1.9		1. Clarithromycin		1. Amiodarone	
30–39 years	339 (34.8)	1. Do you review all medications that atorvastatin-treated patients are currently using?	742 (76.3)	231 (23.7)	Low	366 (37.6)	Low	416 (42.8)
40–49 years	199 (20.5)	2. Do you target patients who are prescribed several medications concurrently (i.e., polypharmacy) as a specific population of concern for drug interactions?	779 (80.1)	194 (19.9)	Intermediate	161 (16.5)	Intermediate	231 (23.7)
50–59 years	78 (8)	3. Do you seek non-drug alternative treatment options initially?	501 (51.5)	472 (48.5)	High	446 (45.8)	High	326 (33.5)
60 years or more	24 (2.5)	4. Do you start therapy with only one medication at a time, when it is possible?	800 (82.2)	173 (17.8)	2. Cyclosporine		2. Digoxin	
Gender		5. Do you educate patients about possible atorvastatin-induced adverse reactions to ensure early recognition?	828 (85.1)	145 (14.9)	Low	286 (29.4)	Low	335 (34.4)
Male	710 (73)	6. Do you encourage patients to always read labels of OTC medications carefully and learn about any precautions or major DDIs linked to Atorvastatin?	678 (69.7)	295 (30.3)	Intermediate	207 (21.3)	Intermediate	227 (23.3)
Female	263 (27)	7. Do you ask the patient about familial responses to atorvastatin or relevant medications?	678 (69.7)	295 (30.3)	High	480 (49.3)	High	411 (42.2)
Governorate		8. Do you educate yourself about new medications and indications from up-to-date, reliable web-based information sources like Medscape, Drugs.com, or FDA.gov?	858 (88.2)	115 (11.8)	3. Erythromycin		3. Azithromycin	
Suez canal region	471 (48.4)	9. Do you use screening tools such as the Drug Associated Risk Tool (DART) to identify patients at risk of Atorvastatin drug interactions?	449 (46.1)	524 (53.9)	Low	383 (39.4)	Low	318 (32.7)
Greater cairo region	217 (22.3)	10. Are you familiar with the pharmacokinetics of Atorvastatin and other drugs that are commonly prescribed in combination?	691 (71)	282 (29)	Intermediate	281 (28.9)	Intermediate	261 (26.8)
Delta region	193 (19.8)				High	309 (31.8)	High	394 (40.5)
Upper Egypt Region	47 (4.8)				4. Fenofibrate		4. Pectin	
Alexandria region	45 (4.6)				Low	474 (48.7)	Low	590 (60.6)
Educational background					Intermediate	207 (21.3)	Intermediate	165 (17)
BSc	649 (66.7)				High	292 (30)	High	218 (22.4)
Diploma	165 (17)				5. Fluconazole		5. Clopidogrel	
MSc	70 (7.2)				Low	347 (35.7)	Low	431 (44.3)
Ph.D.	89 (9.1)				Intermediate	269 (27.6)	Intermediate	261 (26.8)
Years of experience in community pharmacies					High	357 (36.7)	High	281 (28.9)
< 5	207 (21.3)				6. Gemfibrozil		6. Phenytoin	
5–10	306 (31.4)				Low	391 (40.2)	Low	378 (38.8)
10–15	186 (19.1)				Intermediate	254 (26.1)	Intermediate	196 (20.1)
16–20	123 (12.6)				High	328 (33.7)	High	399 (41)
> 20	151 (15.5)				7. Ketoconazole		7. Fluvoxamine	
Number of patients encountered per day					Low	387 (39.8)	Low	454 (46.7)
< 50	205 (21.1)				Intermediate	223 (22.9)	Intermediate	266 (27.3)
50–100	378 (38.8)				High	363 (37.3)	High	253 (26)
101–150	187 (19.2)				8. Leflunomide			
151–200	102 (10.5)				Low	509 (52.3)		
> 200	101 (10.4)				Intermediate	224 (23)		
Information resource used to check DDIs (n = 972; one missing response)					High	240 (24.7)		
Internet or mobile apps	530 (54.5)				9. Nefazodone			
Medical textbooks	288 (29.6)				Low	530 (54.5)		
Medical journals	49 (5)				Intermediate	230 (23.6)		
Package leaflets	105 (10.8)				High	213 (21.9)		
					10. Niacin			
					Low	363 (37.3)		
					Intermediate	284 (29.2)		
					High	326 (33.5)		

*Cumulative role scores are represented as the means ± SDs; CP, community pharmacist; pDDIs: potential drug‒drug interaction; ATV, atorvastatin; OTC, over-the-counter.

### Pharmacists’ roles in dispensing and monitoring atorvastatin

**Fig. 1:**
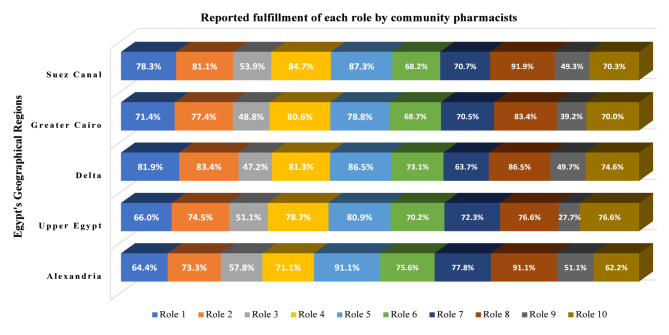
Proportions of pharmacists (categorized by geographical region) performing each of the listed roles. Statistically significant regional differences were observed for Roles 5, 8, and 9 (see Table 2 for the chi-square results). Role 1: Reviewing all the medications used by atorvastatin-treated patients; Role 2: Targeting patients who are prescribed multiple concurrent medications (polypharmacy) as a population at increased risk of drug–drug interactions; Role 3: seeking nondrug alternative treatment options initially; Role 4: Initiate therapy with a single medication when possible; Role 5: educating patients about potential atorvastatin-induced adverse reactions to facilitate early recognition; Role 6: encouraging patients to read over-the-counter (OTC) medication labels to identify precautions or major atorvastatin-related DDIs; Role 7: inquiring about familial responses to atorvastatin or related medications; Role 8: using up-to-date, reliable web-based information sources (e.g., Medscape, Drugs.com, FDA.gov); Role 9: use of structured screening tools such as the drug-associated risk tool (DART) to identify patients at risk of atorvastatin drug interactions; Role 10: Being familiar with the pharmacokinetics of atorvastatin and commonly coprescribed medications.

The mean cumulative role-fulfillment score was 7.2 ± 1.91, indicating generally high engagement in recommended practices. The frequencies for each role are presented in Table 1. The most frequently performed roles included using reliable web-based resources (88.2%), counseling patients about ATV-related ADRs (85.1%), initiating single-drug therapy when possible (82.2%), and targeting patients with polypharmacy (80.1%). The least commonly practiced behaviors were the use of structured screening tools such as DART (46.1%), followed by the recommendation of nonpharmacological options as a first approach (51.5%). These regional trends are illustrated in Fig. 1. Although Fig. 1 provides a descriptive overview of regional variations in pharmacists’ roles, formal statistical testing indicated that most roles were performed at broadly comparable levels across regions. Significant regional differences were observed for selected roles only, with counseling patients about ATV-related ADRs (role 5), the use of web-based drug-information resources (role 8), and the use of structured screening tools such as DART (role 9) being reported more frequently in the Suez Canal region than in Greater Cairo and/or Upper Egypt (Table 2).

**Table 2:** Association studies of pharmacists’ roles by gender, educational level and information resources.

Variables	Gender			Educational background					Information resource used to check DDIs				
	Male	Female	*p* value	BSc	Diploma	MSc	Ph.D.	*p* value	Internet/Mobile apps	Medical journals	Medical textbooks	Package leaflets	*p* value
Cumulative role scores*	7.08 ± 1.9	7.52 ± 1.6	0.016^1^	7.21 ± 1.88	7.47 ± 1.81	6.9 ± 2.3	6.82 ± 1.87	0.026^2^	7.1 ± 2.03	7.04 ± 1.96	7.34 ± 1.73	7.38 ± 1.68	0.416^2^
				Post hoc: Ph.D.—Diploma (adj. * p* = 0.02)									
Role 1													
Yes	76.3	76	0.924	75.8	83	75.7	67.4	0.044	77.3	69.4	74.7	81	0.384
No	23.7	24		24.2	17	24.3	32.6		22.7	30.6	25.3	19	
Role 2													
Yes	78.9	83.3	0.127	80.1	80.6	80	78.7	0.986	80.1	77.6	81.9	69	0.776
No	21.1	16.7		19.9	19.4	20	21.3		19.9	22.4	18.1	31	
Role 3													
Yes	49.6	56.7	0.05	51.8	51.5	51.4	49.4	0.982	48.7	59.2	53.5	69	0.002
No	50.4	43.3		48.2	48.5	48.6	50.6		51.3	40.8	46.5	31	
Role 4													
Yes	80.7	86.3	0.042	83.2	84.2	78.6	74.2	0.139	81	81.6	84.4	85.7	0.131
No	19.3	13.7		16.8	15.8	21.4	25.8		19	18.4	15.6	14.3	
Role 5													
Yes	83.2	90.1	0.007	85.4	89.7	82.9	76.4	0.039	85.2	83.7	86.5	76.2	0.301
No	16.8	9.9		14.6	10.3	17.1	23.6		14.8	16.3	13.5	23.8	
Role 6													
Yes	67.6	75.3	0.021	72.4	66.7	57.1	65.2	0.028	70.7	53.1	71.2	64.3	0.012
No	32.4	24.7		27.6	33.3	42.9	34.8		29.3	46.9	28.8	35.7	
Role 7													
Yes	67.7	74.9	0.031	69.6	73.3	65.7	66.3	0.563	69	65.3	70.8	76.2	0.451
No	32.3	25.1		30.4	26.7	34.3	33.7		31	34.7	29.2	23.8	
Role 8													
Yes	88.6	87.1	0.514	87.8	89.7	84.3	91	0.543	87	87.8	90.3	90.5	0.564
No	11.4	12.9		12.2	10.3	15.7	9		13	12.2	9.7	9.5	
Role 9													
Yes	45.4	48.3	0.414	43.9	49.1	51.4	52.8	0.235	46	44.9	45.8	52.4	0.868
No	54.6	51.7		56.1	50.9	48.6	47.2		54	55.1	54.2	47.6	
Role 10													
Yes	69.9	74.1	0.191	71.2	79.4	62.9	60.7	0.006	68.5	81.6	75	66.7	0.008
No	30.1	25.9		28.8	20.6	37.1	39.3		31.5	18.4	25	33.3	

*Cumulative scores are represented as the mean ± SD; 1: p value for the Mann‒Whitney U test; 2: p value for the Kruskal‒Wallis H test.

#### Group comparisons

The Shapiro–Wilk test indicated deviation from normality (*p* < 0.01). Nevertheless, given the large sample size, cumulative role scores are reported as the means ± SDs for descriptive purposes, whereas nonparametric tests were applied for inferential analyses. Compared with males, females presented significantly higher cumulative role scores (7.52 ± 1.59 vs. 7.08 ± 1.99; Mann–Whitney U, *p* = 0.016). Similarly, diploma holders scored significantly higher than Ph.D. holders did (7.47 ± 1.81 vs. 6.82 ± 1.87; Kruskal–Wallis test, adjusted *p* = 0.02). These results are presented in Table 2.

Consistent findings were observed when nonparametric descriptive measures were used: females presented higher cumulative role scores than males did, with both groups having a median score of 8 but differing in distribution, as reflected by an interquartile range (IQR) of 2. Similarly, diploma holders had higher cumulative role scores than did Ph.D. holders, with median values of 8 (IQR 2) and 7 (IQR 2), respectively. Collectively, these results demonstrate that the observed group differences are robust and persist across both parametric and distribution-independent summary approaches.

Chi-square analyses revealed that several roles (roles 3, 4, 5, 6, and 7) were significantly more often practiced by females (Table 2). Regionally, role 5 was more common in the Suez Canal region than in Greater Cairo (adjusted *p* = 0.043), and role 8 was more common in the Suez Canal region than in Greater Cairo and Upper Egypt (adjusted *p* = 0.008 and 0.006, respectively). Role 9 was also more commonly practiced in the Suez Canal region than in Upper Egypt (adjusted *p* = 0.047). These regional differences are shown in Fig. 1.

Educational background also influenced certain roles: diploma holders performed roles 1 and 5 significantly more frequently than did Ph.D. respondents did (adjusted *p* = 0.027 and 0.028). The respondents with a BSc performed role 6 more often than the MSc holders did (adjusted *p* = 0.045), whereas the diploma holders performed role 10 more often than the MSc and Ph.D. respondents did (adjusted *p* = 0.047 and 0.008). These findings are shown in Table 2.

The respondents relying on package leaflets performed role 3 more frequently than those using internet/mobile applications did (adjusted *p* = 0.003), whereas the app users performed role 10 more frequently than did the respondents depending on medical journals did (adjusted *p* = 0.035). The full subgroup comparisons are presented in Table 2.

### Reported rates of ATV-related drug–drug interactions

#### Major interactions

High-frequency encounters with major ATV-related drug–drug interactions were most frequently reported for cyclosporine (49.3%), clarithromycin (45.8%), ketoconazole (37.3%), fluconazole (36.7%), gemfibrozil (33.7%), niacin (33.5%), erythromycin (31.8%), fenofibrate (30.0%), leflunomide (24.7%), and nefazodone (21.9%) (Table 1).

**Fig. 2:**
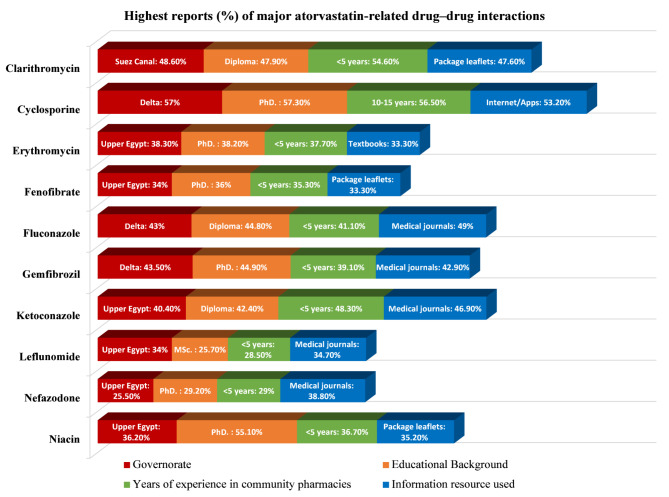
Reported rates of major atorvastatin-related drug–drug interactions across demographic subgroups. For each demographic variable (geographical region, highest academic degree, years of professional experience, and primary drug–drug interaction information resources), only the category with the highest proportion is displayed for visualization. Statistical comparisons across regions were performed via Pearson’s chi-square test. Significant regional differences were observed for cyclosporine (*p* = 0.014), fluconazole (*p* = 0.012), and gemfibrozil (*p* = 0.009). Additional chi-square analyses revealed significant associations between selected demographic characteristics and perceived interaction frequencies, as detailed in the Results section. Bonferroni-adjusted post hoc analyses were conducted only when overall chi-square tests were statistically significant.

The regional distributions of perceived interaction frequencies are illustrated in Fig. 2. Upper Egypt demonstrated descriptively higher proportions of high-frequency interactions involving erythromycin, fenofibrate, ketoconazole, leflunomide, nefazodone, and niacin; however, these regional differences were not statistically significant (*p* > 0.05 for all). In contrast, significant regional variation was observed for cyclosporine (χ^2^ = 19.144, *p* = 0.014), fluconazole (χ^2^ = 19.628, *p* = 0.012), and gemfibrozil (χ^2^ = 20.377, *p* = 0.009). Bonferroni-adjusted post hoc analyses revealed higher intermediate-frequency reporting of cyclosporine in Upper Egypt than in the Suez Canal and Delta regions, higher intermediate-frequency fluconazole reporting in Greater Cairo than in the Suez Canal and Delta regions, and higher high-frequency gemfibrozil reporting in the Delta region than in Greater Cairo.

The perceived frequencies of major ATV-related drug–drug interactions differed significantly according to pharmacists’ educational level, years of professional experience, and information resources used. The highest academic degree was significantly associated with reported interactions involving erythromycin (χ^2^ = 14.273, *p* = 0.027), fluconazole (χ^2^ = 13.820, *p* = 0.032), gemfibrozil (χ^2^ = 12.743, *p* = 0.047), and niacin (χ^2^ = 27.036, *p* < 0.001); post hoc analyses indicated higher niacin interaction frequencies among pharmacists holding doctoral degrees than among those in other educational groups (Bonferroni-adjusted *p* < 0.01), whereas no significant pairwise differences were observed for gemfibrozil. Years of professional experience were associated with perceived interactions for clarithromycin (χ^2^ = 28.351, *p* < 0.001), ketoconazole (χ^2^ = 18.479, *p* = 0.018), and gemfibrozil (χ^2^ = 15.811, *p* = 0.045); post hoc comparisons confirmed experience-dependent differences for clarithromycin and ketoconazole but not for gemfibrozil. Additionally, the primary information resource used to check drug–drug interactions was significantly associated with nefazodone interaction frequency (χ^2^ = 14.479, *p* = 0.025), with pharmacists relying on medical journals reporting higher frequencies than those using other sources (Bonferroni-adjusted *p* < 0.05).

#### Moderate interactions

High-frequency reports for moderate DDIs included amiodarone (33.5%), digoxin (42.2%), azithromycin (40.5%), pectin (22.4%), clopidogrel (28.9%), phenytoin (41.0%), and fluvoxamine (26.0%) (Table 1).

**Fig. 3:**
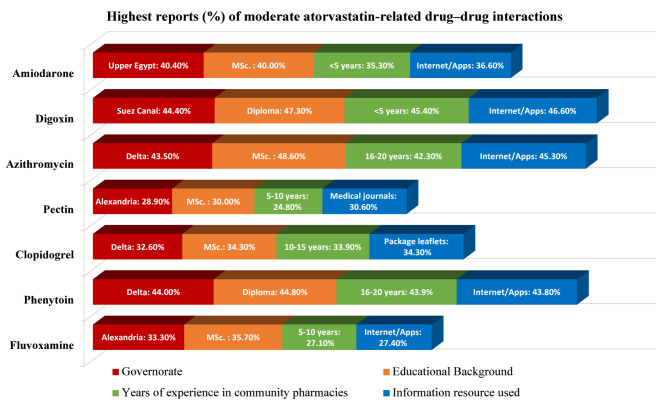
Reported rates of moderate atorvastatin-related drug–drug interactions across demographic subgroups. For each demographic variable (geographical region, highest academic degree, years of professional experience, and primary drug–drug interaction information resources), only the category with the highest proportion is displayed for visualization. Statistical comparisons across regions were performed via Pearson’s chi-square test. A significant regional difference was observed only for digoxin (*p* = 0.035). Additional chi-square analyses revealed significant associations between selected demographic characteristics and perceived interaction frequencies, as detailed in the Results section. Bonferroni-adjusted post hoc analyses were conducted only when overall chi-square tests were statistically significant.

Regional comparisons revealed a significant association only for digoxin, where intermediate-frequency encounters were reported more frequently among pharmacists practicing in Greater Cairo than among those practicing in the Suez Canal region (*p* = 0.035; Bonferroni-adjusted *p* = 0.003). Although respondents from the Delta reported the highest proportions of azithromycin, clopidogrel, and phenytoin interactions, those from Upper Egypt reported the highest rates of amiodarone-related DDIs, and Alexandria demonstrated the highest proportions of pectin and fluvoxamine interactions; these regional differences were not statistically significant (Fig. 3).

Educational level showed a significant overall association only for fluvoxamine reporting (*p* = 0.038); however, post hoc pairwise comparisons did not reveal statistically significant differences between educational groups after Bonferroni adjustment. Descriptively, MSc respondents reported the highest proportions of amiodarone, azithromycin, pectin, clopidogrel, and fluvoxamine interactions, whereas diploma holders reported the highest proportions of digoxin and phenytoin interactions (Fig. 3).

Years of professional experience significantly influenced azithromycin (*p* = 0.014) and clopidogrel (*p* = 0.009) reporting patterns. Pharmacists with more than 20 years of experience reported significantly more low-frequency azithromycin encounters than did those with less than 5 years of experience (post hoc *p* = 0.001), whereas pharmacists with less than 5 years of experience reported more intermediate-frequency encounters (post hoc *p* = 0.006). For clopidogrel, pharmacists with more than 20 years of experience reported significantly more low-frequency encounters than did those with 11–15 years of experience (post hoc *p* < 0.001), whereas pharmacists with 11–15 years of experience reported more intermediate-frequency encounters than did those with more than 20 years of experience (post hoc *p* = 0.044).

The primary information resource used to identify DDIs significantly influenced reporting patterns for digoxin (*p* = 0.022) and azithromycin (*p* = 0.030). Pharmacists relying primarily on internet or mobile applications reported significantly more high-frequency encounters than those relying on package leaflets for both digoxin (post hoc *p* = 0.014) and azithromycin (posthoc *p* = 0.031). The participants who used internet/mobile applications also reported higher encounter frequencies for several additional interactions, including amiodarone, phenytoin, and fluvoxamine, whereas the respondents who relied on medical journals reported the highest proportions of pectin interactions (Fig. 3).

## Discussion

This national survey provides a comprehensive overview of CPs’ engagement with ATV therapy and their real‑world exposure to clinically significant DDIs across Egypt. Although respondents generally demonstrated strong adherence to several recommended practices, the high reported frequencies of both major and moderate DDIs—particularly in Upper Egypt and the Delta—highlight a meaningful gap between best-practice expectations and the practical challenges faced in routine community‑pharmacy settings. These findings are consistent with earlier reports describing variability in patient safety culture within Egyptian healthcare settings^57^, suggesting that additional support structures may be needed to optimize DDI management.

The roles most frequently undertaken by pharmacists, such as obtaining complete medication histories, monitoring polypharmacy, and counseling on adverse effects, are consistent with the evolving clinical responsibilities of CPs in patient-centered pharmaceutical care^58–62^. However, elevated exposure to high-risk ATV combinations reinforces the need to strengthen systematic DDI screening processes in community practice. The prominence of macrolide–ATV interactions is consistent with previously documented inappropriate antibiotic use patterns in Egypt^43,44,63,64^, underscoring the need for collaborative antimicrobial stewardship efforts. Similarly, frequent cyclosporine–ATV interactions may reflect the substantial burden of posttransplant metabolic complications^45–47^ and chronic liver disease nationally^48^, both of which commonly necessitate statin therapy^49–51^.

The high frequency of phenytoin- and digoxin-related interactions corresponds with known comorbidity trends, including dyslipidemia among patients with epilepsy^52^ and the established association between lipid abnormalities and heart failure risk^65,66^. These patterns underscore the need for CPs to maintain heightened vigilance when dispensing statins to patients with multiple chronic conditions.

Pharmacist-related factors also influence DDI detection. Female pharmacists and diploma holders demonstrated greater adherence to recommended practices, reflecting prior findings that female healthcare professionals may employ more patient-centered communication styles and rely more consistently on digital decision-support tools^67–69^. Pharmacists with postgraduate qualifications reported more DDI encounters, in line with earlier evidence suggesting greater guideline awareness among advanced degree holders^70^. While more experienced pharmacists identified more moderate DDIs, less experienced respondents reported higher frequencies of major interactions. This pattern may reflect differing practice environments or knowledge-retention gaps previously noted in the literature^71–74^.

Digital resources, particularly mobile applications, were the most widely used DDI-checking tools across all regions, especially among younger and early-career pharmacists. These resources offer rapid, updated clinical information^75,76^, although variability between decision-support systems has been documented^77^. Pharmacists who frequently used journal articles also reported more DDIs, indicating that multimodal evidence retrieval may increase clinical vigilance. This observation supports expanding access to high‑quality, locally relevant clinical pharmacy decision-support platforms.

### Implications for clinical pharmacy practice

The findings of this study highlight several practice-oriented implications for optimizing ATV safety in community settings. First, standardizing risk stratification through structured tools such as the DART may increase the consistency of identifying patients at high risk for ATV-related drug–drug interactions rather than directly detecting the interactions themselves. Although DART is not a DDI-specific instrument, it systematically flags patients with characteristics known to predispose them to clinically significant DDIs—including polypharmacy, multiple comorbidities, advanced age, and the use of high-risk medications. In community pharmacy practice, DART can therefore be used as an initial screening step to prioritize vulnerable patients for subsequent, focused DDI assessment via validated interaction databases (e.g., Micromedex®, Drugs.com), thereby improving the efficiency and clinical relevance of ATV DDI detection^29,38^. Targeted training for early-career pharmacists is also warranted, particularly to strengthen competence in managing complex statin combinations. Given the disproportionately high DDI frequencies observed in Upper Egypt and the Delta, region-specific educational and stewardship initiatives are needed to support pharmacists practicing in higher-risk environments. Finally, encouraging the combined use of digital decision-support resources alongside peer-reviewed literature may foster more comprehensive and reliable DDI detection in routine clinical practice.

### Future research

Future studies should explore the determinants of regional variation in ATV-related DDIs and assess whether pharmacist-led interventions can reduce clinically significant outcomes. Mixed-method approaches may help identify barriers to the routine use of structured DDI screening tools. Moreover, linking CP-reported DDIs with prescribing patterns, comorbidity data, and local health system capacity may provide a more comprehensive understanding of high-risk therapeutic combinations.

### Strengths and limitations

Key strengths include the large sample size, national coverage, and detailed assessment of both knowledge and observed practice patterns. This study is limited by its cross-sectional design, which precludes causal inference. The data were self-reported and are therefore subject to recall and social desirability bias. Reported ATV-related DDIs reflect pharmacists’ experiential observations rather than verified prescription records or clinical outcomes and should be interpreted as perceived exposure rather than confirmed events. Interaction frequencies were assessed using ordinal categories, limiting the estimation of true incidence or clinical severity. Additionally, unmeasured factors—such as prescriber behavior, patient comorbidity burden, and regional healthcare variability—may have influenced the findings.

### Conclusions

This national cross-sectional survey describes CPs’ practices related to ATV therapy and their self-reported exposure to clinically relevant DDIs in Egypt. Although high adherence to several recommended safety practices was observed, CPs reported frequent encounters with both major and moderate ATV-related DDIs, most commonly involving clarithromycin, cyclosporine, digoxin, phenytoin, and azithromycin. Geographic variation in reported interaction frequencies was evident, with higher proportions observed in Upper Egypt and the Delta.

These findings underscore the role of CPs in identifying potentially high-risk medication combinations during routine dispensing activities. However, the reported interaction frequencies reflect pharmacists’ experiential observations rather than confirmed clinical events and should therefore be interpreted as perceived exposure. The results indicate a need for more standardized approaches to interaction risk identification, improved access to validated decision-support resources, and targeted professional development initiatives, particularly in regions with higher reported interaction exposure. Further research incorporating prescription-level data and clinical outcome verification is required to clarify the clinical relevance of these observations and to assess the effectiveness of pharmacist-led safety interventions.

## Data Availability

To ensure participant confidentiality, the datasets generated and analyzed in this study are not publicly available. However, anonymized data may be provided by the corresponding author upon justified request, subject to ethical approval.
